# Progressive Structural Complexity in Ferroelectric 1,2,4-Triazolium Hexabromoantimonate(III): Interplay of “Order–Disorder” and “Displacive” Contributions to the Structural Phase Transitions

**DOI:** 10.1021/acs.jpclett.3c00924

**Published:** 2023-05-09

**Authors:** Michał Chański, Agata Białońska, Ryszard Jakubas, Magdalena Rok, Jan K. Zaręba, Rafał Janicki, Piotr Durlak, A. Piecha-Bisiorek

**Affiliations:** † Faculty of Chemistry, University of Wrocław, Wrocław 50-383, Poland; ‡ Institute of Advanced Materials, Faculty of Chemistry, Wrocław University of Science and Technology, Wrocław 50-370, Poland

## Abstract

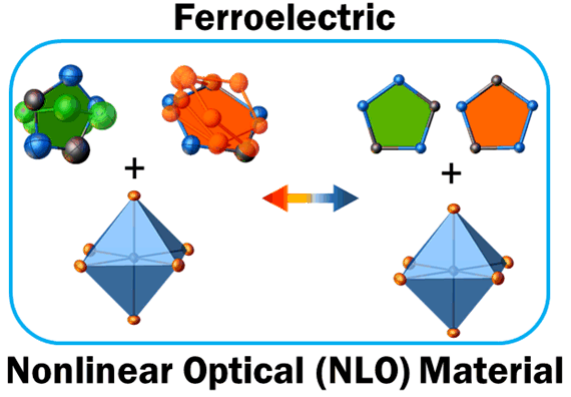

Halobismuthates(III) and haloantimonates(III) with the R_3_MX_6_ chemical composition create a new and broadly unexplored class of ferroelectric compounds. In this paper, we report the haloantimonate(III) ferroelectric comprising an aromatic (1,2,4-triazolium) cation, i.e., (C_2_N_3_H_4_)_3_[SbBr_6_] (**TBA**). Temperature-resolved structural and spectroscopic studies indicate that **TBA** undergoes two solid–solid phase transitions between tetragonal [*P*4_2_/*m* (I)] and monoclinic [*P*2_1_/*n* (II) and *P*2_1_ (III)] phases. **TBA** experiences a paraelectric–ferroelectric phase transition at 271/268 K (II–III) driven by “order–disorder” and “displacive” molecular mechanisms. The ferroelectric properties of phase III have been confirmed by hysteresis loop measurement, and additionally, the acentric order has been further supported by second-harmonic generation measurements. Insight into the molecular origins of the ferroelectric polarization was provided by periodic *ab initio* calculations using the Berry phase approach at the density functional theory (DFT-D3) method level employed for calculations of spontaneous polarization.

Hybrid organic–inorganic perovskites based on Sn(II), Ge(II), and Pb(II) have emerged as novel functional materials that arouse particular interest in energy storage, energy conversion, and light-emission applications.^1−7^ An undesirable feature of these materials is chemical instability and environmental toxicity, both of which prevent widespread adoption and commercialization. In this context, the Bi(III) and Sb(III) species provide alternative synthetic possibilities of novel lead-free hybrid perovskites, owing to their isoelectronic (6s^2^/5s^2^) configuration with Pb(II), their being much more environmentally benign, and their good chemical stability.^8^

In particular, underexplored groups of haloantimonates(III) and halobismutates(III) with the R_*a*_M_*b*_X_3*b*+*a*_ general formula [where R is an organic cation, M = Sb(III) or Bi(III), and X = Cl, Br, or I] have become the focus of interest because they feature easy synthesis and processing, are inexpensive, and exhibit relevant electrical and optical properties, such as ferroelectricity and light emission.^9−12^ These molecular–ionic materials are characterized by a significant diversity of anionic networks [from zero-dimensional (0D) to one-dimensional (1D), two-dimensional (2D), or even three-dimensional (3D) architectures].^13−17^ Interestingly, the ferroelectric properties are found for a limited number of stoichiometric types and selected anionic forms such as R_5_M_2_X_11_ (0D),^18^ R_3_M_2_X_9_ (0D and 2D),^19,20^ RMX_4_ (1D),^21^ R_2_MX_5_ (1D),^22,23^ and R_3_MX_6_ (0D).^24^

Haloantimonates(III) and halobismuthates(III) with isolated/discrete [MX_6_]^3–^ moieties in the crystal structure are quite common; nevertheless, only a few representatives have been tested for temperature-induced structural phase transitions (PTs) and piezoelectric and ferroic properties.^25−27^ Of 240 0D compounds that adapted the R_3_MX_6_ stoichiometry, only 42 were found to crystallize in the acentric space group.

Recently, 0D (R_3_MX_6_-type) bromobismuthate(III) characterized by a two-component cationic network has been reported by Wang et al.^24^ The compound described by the formula [((CH_3_)_2_NH_2_)(C_6_H_5_CH_2_NH_3_)_2_][BiBr_6_] contains two benzylammonium cations, one dimethylammonium molecule, and discrete [BiBr_6_]^3–^ moieties. This compound exhibits room-temperature multiaxial ferroelectric properties with a *P*_s_ of 1.0 μC cm^–2^ (Scheme 1).

**Scheme 1:**
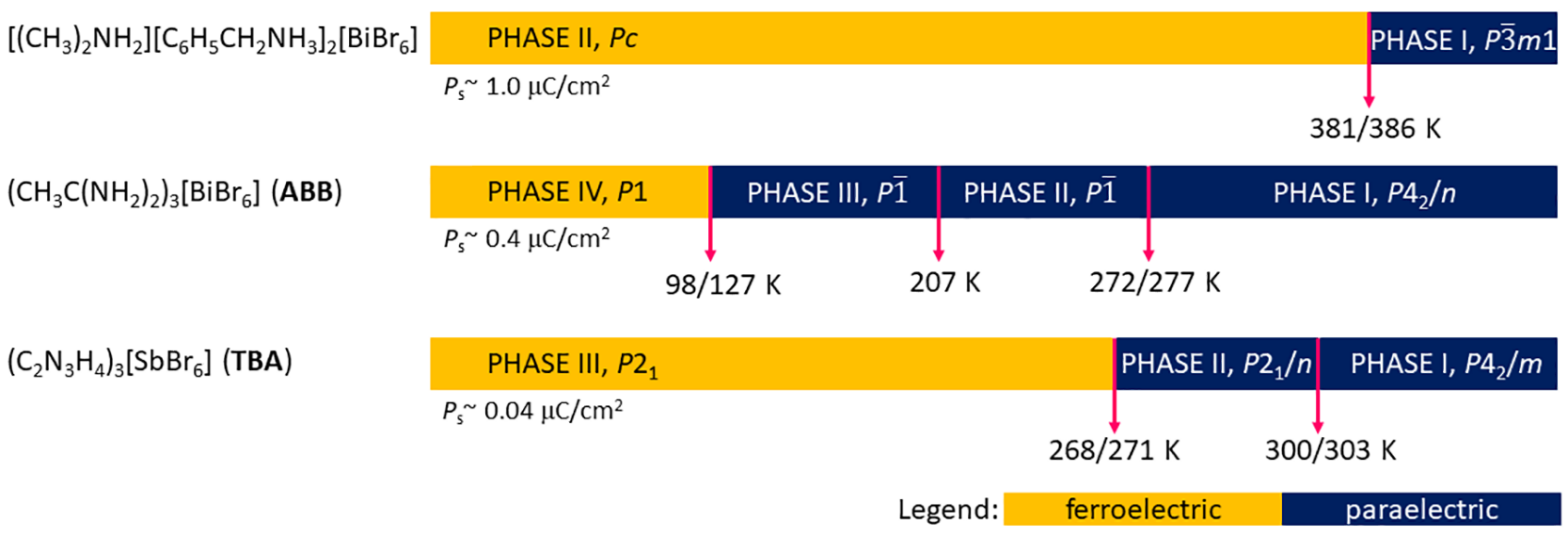
Phase Diagrams of R_3_MX_6_ Ferroelectrics

Lately, we have successfully synthesized and characterized the first ferroelectric of the halobismuthate(III) family with an R_3_MX_6_ stoichiometry, that is, tris(acetamidinium) hexabromobismuthate(III), (CH_3_C(NH_2_)_2_)_3_[BiBr_6_] (ABB), having a one-component organic network.^28^ Rich polymorphism in the solid state of ABB occurs between tetragonal (paraelastic) and triclinic (ferroelastic) crystal phases: I (*P*4_2_/*n*) → II (*P*1̅) at 272/277 K (cooling/heating), II (*P*1̅) → III (*P*1̅) at 207 K, and III (*P*1̅) → IV (*P*1) at 98/127 K (Scheme 1). The ferroelectric phase was confirmed for phase IV, whereas the antiferroelectric arrangement is suggested to appear in phase III. The ferroelectric transition (III → IV) should be considered as “displacive” for both cationic and anionic substructures.

As one can see, previous reports on ferroelectrics with the R_3_MX_6_ chemical stoichiometry provided a new perspective for obtaining ferroelectric materials belonging to the groups of haloantimonates(III) and halobismutates(III). Stimulated by the literature, we employed a simple, small aromatic heterocyclic building block (1,2,4-triazolium), which is practically unexplored in assemblies with halometals(III), and managed to obtain a new representative of the family of R_3_MX_6_ haloantimonates(III), namely, (C_2_N_3_H_4_)_3_[SbBr_6_] (**TBA**).

**TBA** was synthesized from a mixture of 1,2,4-triazole, SbBr_3_, and concentrated HBr in methanol [more details in Sections 1 and 2 of the Supporting Information (Figure S1)]. **TBA** is stable up to ∼450 K (Figure S2) and undergoes two reversible PTs at 303/300 K (heating/cooling) and 271/268 K, both of a discontinuous nature (Figure S3). The transition entropies (Δ*S*_tr_) accompanying these PTs were found to be significant: 9.2 and 10.3 J mol^–1^ K^–1^, respectively. This indicates the “order–disorder” character of the molecular mechanism.

Above 303 K, **TBA** belongs to space group *P*4_2_/*m* of the tetragonal system (Table S1). In an asymmetric part of the unit cell, there are two triazolium cations (A and B) and the Br–Sb–Br fragment of the [SbBr_6_]^3–^ anion (Figure 1a). The Sb atom of the [SbBr_6_]^3–^ anion is located at a special position (2/*m*..; coordinates of ^1^/_2_, 0, 0). The bromine atoms form a slightly distorted octahedral environment for the Sb1 atom (Table S2).

**Figure 1:**
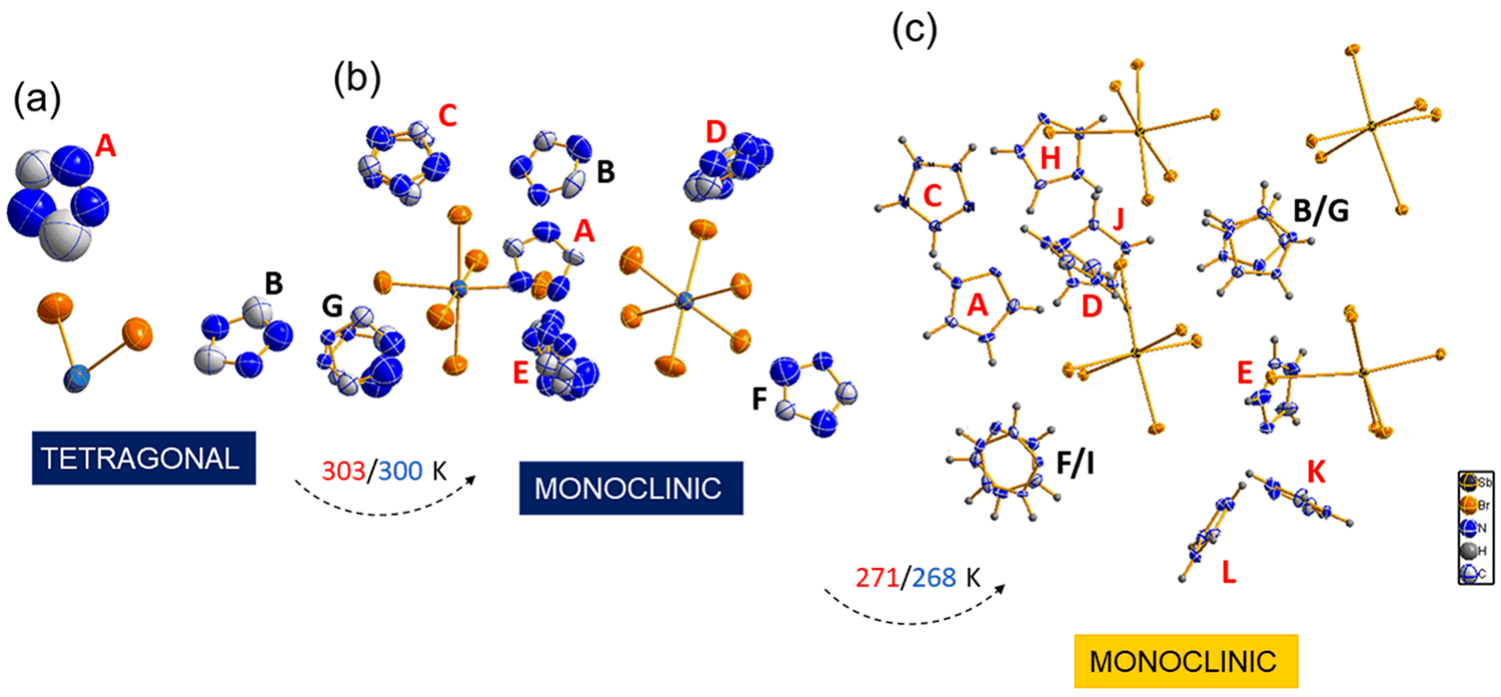
Asymmetric part of the unit cell of **TBA** at (a) 320 K (phase I), (b) 293 K (phase II), and (c) 100 K (phase III). The cations of the **a** and **b** groups are colored red and black, respectively.

The Br1 and Br3 atoms are located at the axial and equatorial positions, respectively, of the octahedral unit. The octahedral anionic units are arranged in double helices. The triazolium cations reveal dynamic disorder, making the distinction between carbon and nitrogen sites impossible. The center of gravity of cations A and B is located at a special position, *m*... for cations A and 2/*m*.. (coordinates of 0, 0, 0) for cations B (Figure S4).

Similar to the anionic units, cations A are arranged as double helices. Bromine atoms Br1 from the axial position, being directed toward channels occupied by cations A, separate the cations A from each other along the [001] direction. Unlike cations A, cations B are arranged in stacks extended along the [001] direction. Taking into account the special positions and the atomic displacement parameters, it seems that through rotation the cations A can take any orientation, while the cations B rotate (approximately) in the plane of the triazolium ring.

At 293 K (phase II), **TBA** belongs to space group *P*2_1_/*n* of the monoclinic system. The PT is accompanied by a change in the orientation and dimension of the unit cell. In the centrosymmetric monoclinic phase, lattice constant *b* corresponds to 2*c* of the tetragonal phase, and lattice constants *a* and *c* of the monoclinic phase correspond to the [110] and [−110] diagonals, respectively, of the tetragonal phase (Figure S5). The asymmetric unit consists of two [SbBr_6_]^3–^ anions and seven triazolium cations (Figure 1b). Bromine atoms form a distorted octahedral environment around atoms Sb1 and Sb2. The distortion involves Sb–Br distances as well as Br–Sb–Br angles (Table S2). Three bromine atoms that are *cis*-oriented in relation to each other form short Sb–Br bonds, while the other bromine atoms, also *cis*-oriented to each other, form long Sb–Br bonds. The strongest Sb–Br distortion (shortening and elongation) is observed for bromine atoms occupying axial positions. The Sb–Br distortion causes the Sb atoms of consecutive anionic units to be not in line along the [010] direction (Figure S6). Similar to the tetragonal phase, also in the monoclinic centrosymmetric phase, cations can be divided into two groups, corresponding to cations A and B of the tetragonal phase. Specifically, in the monoclinic centrosymmetric phase, there are four crystallographically unrelated cations (cations A and C–E) that belong to group **a** and three crystallographically unrelated cations (B, F, and G) that belong to group **b** (cations F and G are located in the special position). A majority of the cations (except cations A and B) reveal dynamic disorder that makes the N–C distinction impossible. In group **b**, cations B, F, and G are roughly parallel with each other. The distance between the planes of the triazole rings is <4 Å. The disorder results from the rotation in the plane of the triazole ring. In group **a**, the disorder also results in the rotation in the plane of the triazole ring; however, only cations A and C are approximately parallel-oriented in relation to each other, and the orientation of the rest of the cations in this group is far from parallel.

Further cooling of **TBA** results in one more PT. At 100 K (phase III), the parameters of the unit cell are relatively similar to those at 293 K; however, at 100 K, TBA belongs to space group *P*2_1_ of the monoclinic system. There are four [SbBr_6_]^3–^ units and 12 triazolium cations in an asymmetric part of the unit cell (Figure 1c). Bromine atoms form a distorted octahedral environment around Sb atoms (Table S2). However, the scheme of the short and long Sb–Br bonds differs from that found at 293 K. At both temperatures, the Sb–Br(axial) bonds are almost perpendicular to the 2_1_-fold screw axis. The difference is observed among Sb–Br(equatorial) bonds at 293 and 100 K. At 293 K, along the screw 2_1_-fold axis, the short Sb–Br(equatorial) bonds are directed in one direction and the long Sb–Br(equatorial) bonds are directed in the opposite direction. Because of the symmetry, along an equivalent 2_1_-fold screw axis the orientation of the short and long Sb–Br(equatorial) bonds is opposite. At 100 K, a similar orientation of the short and long Sb–Br(equatorial) bonds is observed for only one crystallographically unrelated [SbBr_6_]^3–^ unit, [Sb(3)Br_6_^3–^] (Figure S7), which contributes to the polarization of the monoclinic noncentrosymmetric phase. With respect to the cations, in a manner similar to that of higher-temperature phases, these can be divided into two groups, **a** and **b**. Group **a** comprises cations denoted as A, C–E, H, and J–L, and group **b** includes cations B, F, G, and I. All of the cations are ordered at 100 K. The cations belonging to group **b** participate in π···π stacking extending along the [010] direction and form N–H···Br and N–H···N hydrogen bonds with the anion and the cations of group **a**, respectively. The N–H···N hydrogen bonds link three consecutive triazolium cations into discrete chains, D–C–B, F–H–A, and K–J–I (or the N–H···N and the C–H···N: L-E-G). Among the discrete chains, consecutive F–H–A discrete chains are linked by C–H···N hydrogen bonds into a one-dimensional chain extending along the [100] direction (Table S3 and Figure S8).

The PT from the tetragonal to the monoclinic centrosymmetric phase involves mainly the displacement of the Sb atoms, leading to shortening and elongation of the Sb–Br bonds. Simultaneously, the partial ordering of the cations is observed. Although positions of the H atoms could not be determined, if one takes into account the shortest N···Br distances, it seems that the ordered cations form N–H···Br hydrogen bonds once PT commences. Further cooling of **TBA**, which leads to the PT from the monoclinic centrosymmetric to the monoclinic polar phase, results in the ordering of the remaining cations, combined with the formation of hydrogen bonds. The PT is also related to the displacement of the Sb atoms, leading to the reorganization of the short and long Sb–Br bonds. Both cation ordering and Sb displacement are the origins of the overall non-zero net dipole moment (Figure 2 and Figure S7). At 100 K, a majority of the crystallographically unrelated cations have their dipole moments oriented almost perpendicular to the [010] direction, and hence, the cations make a small contribution to the polarization. However, there are two crystallographically unrelated cations, E and G with dipole moments oriented nonperpendicular to the [010] direction, and they contribute to the polarization to a much greater extent (Figure 2a,b). The shortening and elongation of the Sb–Br bonds result in induction of the dipole moment for the anions. However, the dipole moment for three crystallographically unrelated anions is oriented approximately perpendicular to the [010] direction, which means that they do not contribute to the polarization. In turn, the orientation of the dipole moment of the remaining crystallographically unrelated anions [Sb(3)Br_6_]^3–^ is nonperpendicular to the [010] direction, and hence, they make a greater contribution to the bulk polarization.

**Figure 2:**
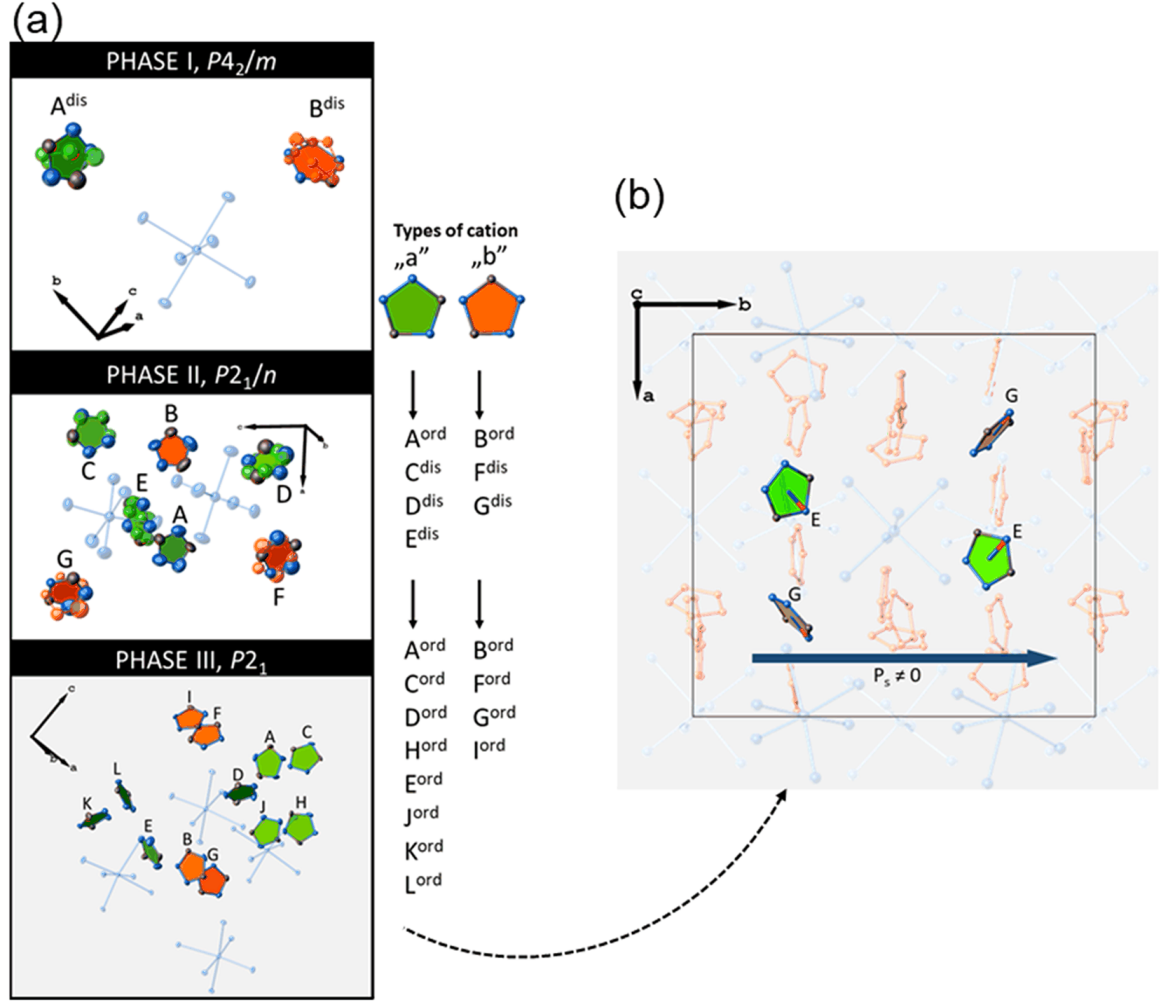
(a) Evolution of the cationic network during PTs. (b) Mutual orientation of the dipole moments contributing to *P*_s_ within the cationic network.

Irradiation of **TBA** with femtosecond laser pulses (1300 nm) in the temperature range of 234–318 K has been employed to provide independent proof of the structural noncentrosymmetry of phase III. The temperature plot of the integral intensities of SHG signals (650 nm) is shown in Figure 3a, and experimental spectra are provided in Figure S9. Data for the cooling and heating cycles attest to the fact that high-temperature phase I and intermediate phase II show no SHG response, yet below approximately 268 K, a clear SHG signal is registered. Accordingly, the observation of SHG in this temperature range confirms our notion that only low-temperature phase III of **TSB** is noncentrosymmetric.

**Figure 3:**
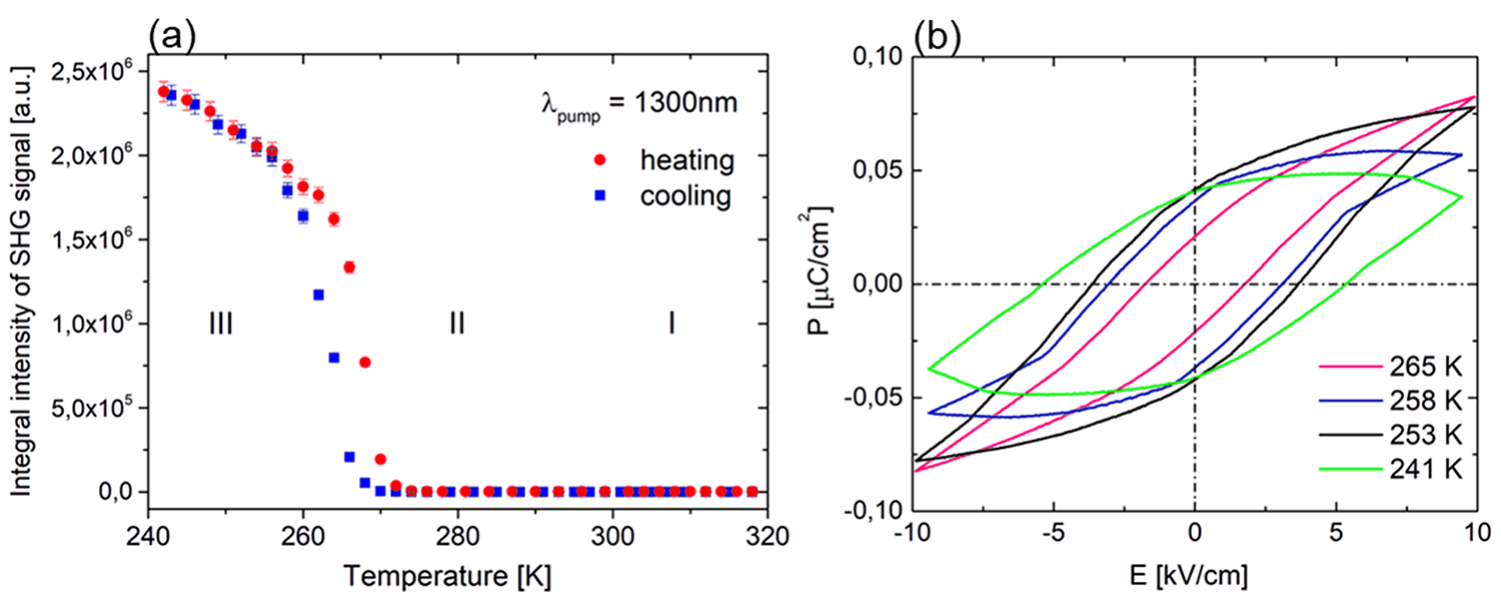
(a) Temperature dependence of SHG for **TBA**. (b) *P*–*E* hysteresis loops observed at various temperatures (sample thickness = 0.8 mm; *f* = 50 Hz).

In the ferroelectric phase, space group *P*2_1_ allows the **TBA** crystal to exhibit spontaneous polarization along the polar *b* axis. The reversal of polarization was investigated on a polycrystalline pellet capacitor with a Au/TBA/Au configuration. As shown in Figure 3b, the *P*–*E* hysteresis loops for selected temperatures prove the ferroelectric properties of **TBA**. Near PT from the paraelectric (II) to ferroelectric (III) phase, the polarization can be reversed under an *ac* of 3.1 kV cm^–1^ at a frequency of 50 Hz. The obtained *P*_s_ value was found to be ∼0.02 μC cm^–2^ at a temperature 1 K below *T*_c_ (265 K). However, at a temperature below 258 K, the *P*_s_ value settled at 0.04 μC cm^–2^ and is roughly constant when *T* – *T*_c_ < 27 K. As the temperature decreases, the coercive field increases slightly to 3.6 and 5.3 kV cm^–1^ for temperatures of 253 and 241 K, respectively.

The dielectric characteristics covering two PTs are presented in panels a and b of Figure 4. The I ↔ II PT is manifested as a distinct step-like anomaly in the curve of the electric response (ε′) with a dielectric increment (Δε) of ≈20, which is quite visible at higher frequencies. The II → III ferroelectric transition is not accompanied by a peak on the ε′(*T*) curve. It is expected to appear below the hertz frequency region (>100 Hz) due to the evident strong relaxation process. At 2 MHz close to 270 K, the dielectric increment (Δε ≈ 10 units) reflects well the ferroelectric PT. The dielectric response seems to be complex because around I → II and II → III PTs one can observe different low-frequency relaxation processes that overlap each other. The higher-frequency process is seen within phases I and II. In turn, over intermediate phase II, the lower-frequency relaxation seems to exhibit critical slowing approaching the ferroelectric PT. The macroscopic relaxation time reaches ∼1 × 10^–4^ s close to *T*_c_. The estimated activation energy (*E*_a_) based on the temperature characteristic of the microscopic time (τ_o_ vs 1/*T*) is not constant over phase II. However, when approaching the critical temperature (*T*_c_ = 271 K), one obtains a value of 6.4 kJ mol^–1^. A detailed analysis of the relaxation processes (Cole–Cole diagrams) within paraelectric phase II is given in Section 6 of the Supporting Information.

**Figure 4:**
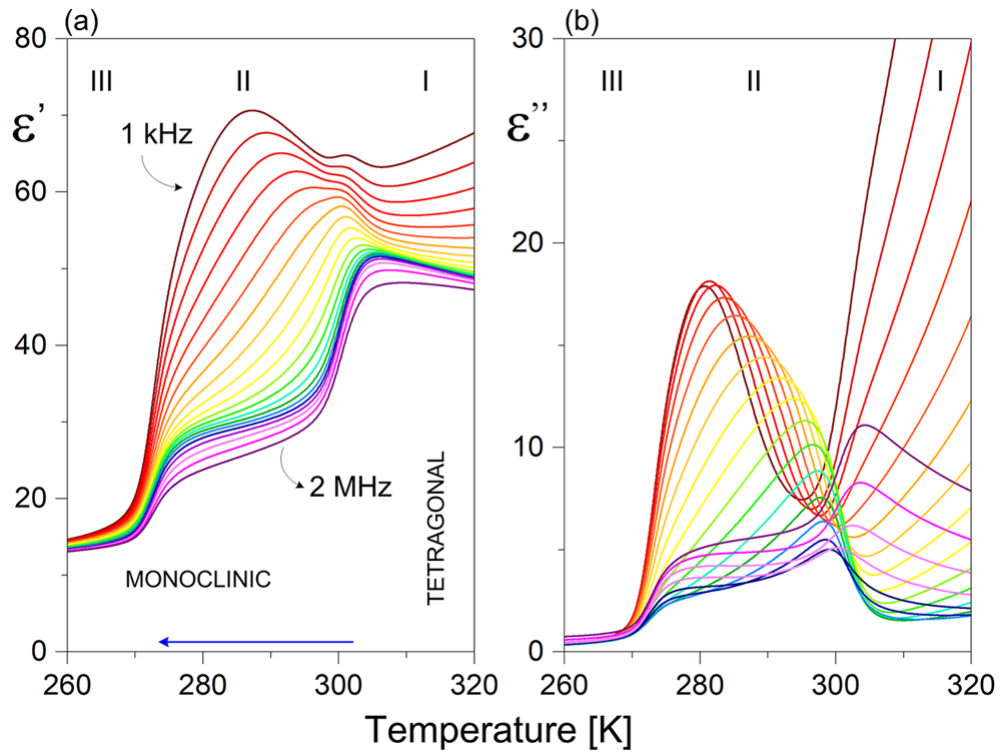
Temperature dependence of the (a) real and (b) imaginary parts of the complex dielectric permittivity of **TBA** during a cooling cycle (pellet sample).

For comparison, we have also performed *ab initio* simulations in the condensed phase for the crystal **TBA** to determine the value of spontaneous polarization. This parameter was calculated and evaluated through a Berry phase (BP) approach^29−31^ as the polarization difference between one of the two enantiomorphic structures (λ = 1 or λ = −1) and the intermediate geometric structure (λ = 0). According to the theory, the macroscopic polarization is best defined (and calculated) as a Berry phase of the electronic Bloch wave functions. In this work, the BP approach at the DFT-D3 method level was used for *P*_s_ calculations. The simulation for *P*_s_ was carried out with the hybrid functional with dispersion correction (B3LYP-D3) coupled with the all-electron basis set (pob_DZVP_rev2, except the combined with ECP basis set for the antimony atom Sb_pob_DZVP_2018) as for the optimization of the structure and lattice parameters. For more details about the calculations, see the Computational Methods of the Supporting Information. Note that BP is a quite sophisticated model because *P*_s_ is calculated directly from the electronic Hamiltonian, as derived from BP theory.^29−31^ This model has been implemented within the CRYSTAL17 software HF framework by Dall’Olio and co-workers in a study of the KNbO_3_ perovskite crystals.^30^ The value of *P*_s_ calculated by us on the basis of quantum theory is 0.059 μC cm^–2^, which is in good agreement with experimental data designated at ∼0.04 μC cm^–2^. This is a good quality result because it must be remembered that in the experiment it was not possible to saturate the polarization of the sample due to crystal breakage.

During the simulation of the crystal **TBA**, the density of states (DOS), full and projected onto molecular orbitals of selected atoms, and the electronic band structure (EBS) were also generated along with the band gap value, which is 3.32 eV (see Figure 5a,b) and is in good agreement with the band gap value 2.96 eV derived from the ultraviolet–visible (UV–vis) spectrum for the **TBA** crystal (more in Section 7 of the Supporting Information). The SeeK-path program was used to determine the k-points along a path within the first Brillouin zone, including the surface in reciprocal space. Usually, the computationally estimated energy gap for semiconductors is slightly overestimated at the DFT level. After many tests, due to the unsatisfactory results of the calculations for the band gap value at the level of B3LYP-D3/POB_DZVP_rev2 basis sets, we used different basis sets and density functionals for every atom, but only for the electronic band gap simulation. Therefore, we used basis sets^32−34^ proposed by Dovesi: 5-11G* for the hydrogen atom, 6-21G* for the carbon and nitrogen atoms, and DURAND-21d1G for the antimony atom. For the bromine atoms, we have used the effective core pseudopotential parameters and corresponding optimized valence basis sets proposed by the Stuttgart/Cologne Group (ECP28MDF).^35^ As mentioned for the full analysis, we have used different density functionals, namely, the range-separated hybrid functional and the screened-Coulomb PBE functional combined with PBE correlation (HSE06-D3).^36,37^ The simulation results of the band gap are very satisfactory for the HSE06-D3 functional, and it must be said that this functional reproduced the experimental value of the band gap very well.

**Figure 5:**
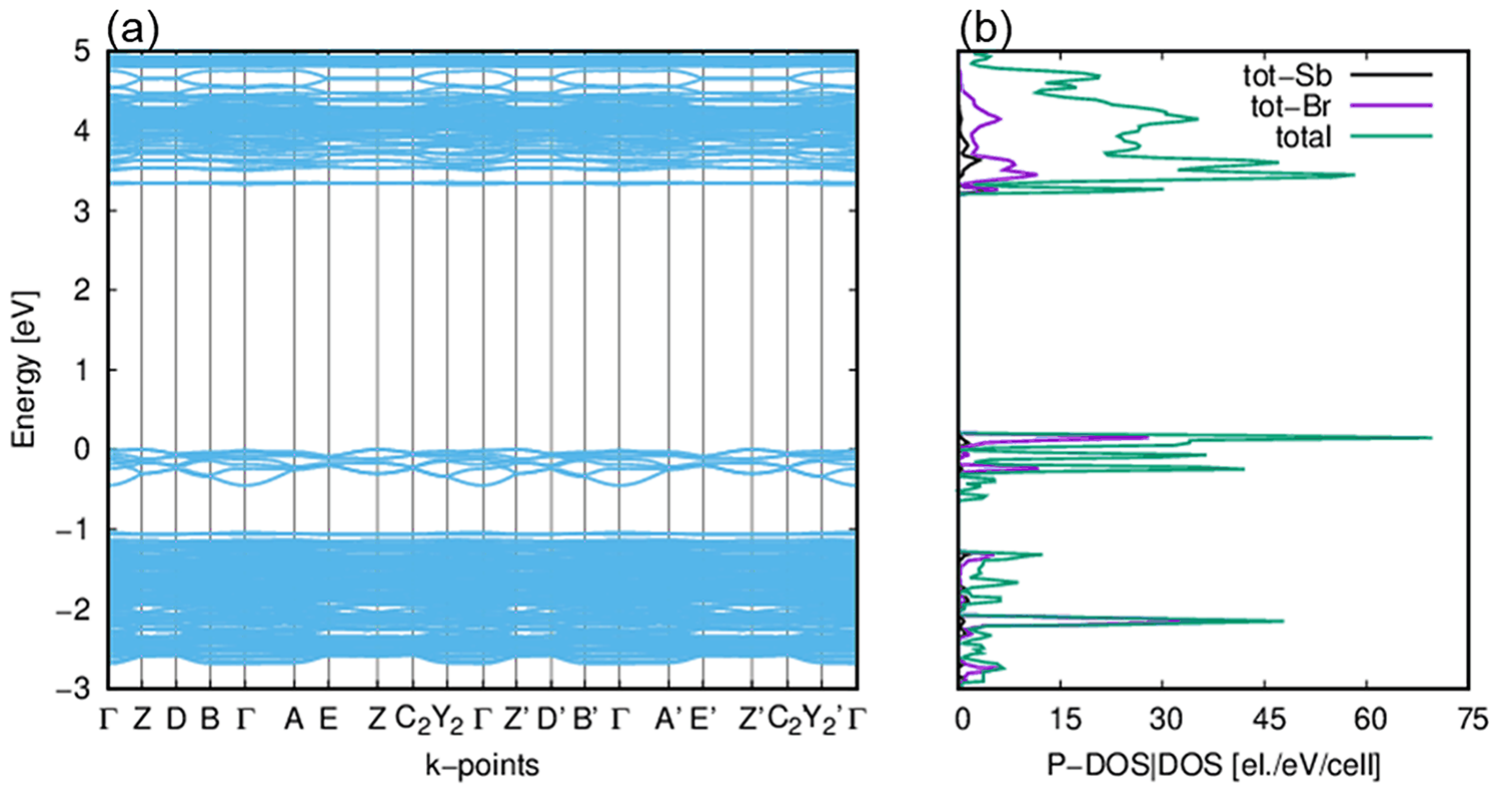
(a) Electronic band structure and (b) density of states of **TBA** derived from the *ab initio* calculations.

Also, the UV–vis–near-infrared absorption and diffuse reflectance spectra were recorded. At least three partially overlapping bands centered at 380, 330, and 250 nm may be distinguished in spectra at room temperature (RT) (Figure S14). Analysis of the recorded spectra may be performed within MO theory.^38,39^ The approximate symmetries of the molecular [SbBr_6_]^3–^ anions in phases I–III are *O*_*h*_, *C*_3*v*_, and *C*_4*v*_, respectively. The geometrical distortion of the [SbBr_6_]^3–^ complex, caused by a decrease in temperature (I → II), leads to stabilization of the occupied a_1_ and unoccupied a_1_ orbitals. Simultaneously, the metal-centered e orbitals are slightly destabilized.^39,40^ Thus, the observed bands in RT absorption spectra of phase II may be attributed to the ^1^A → ^1^A and ^1^A → ^1^E transitions. Due to the presence of two symmetrically independent [SbBr_6_]^3–^ anions (phase II), the aforementioned bands are broadened and not well separated; moreover, these and additional LMCT bands may overlap, as the optical electronegativity difference of Sb(III) and Br(I) ions is ∼0.9. According to the Jørgensen relation,^41^ the LMCT absorption bands in the spectra of antimony(III) bromide systems may occur at approximately 350–400 nm. It is worth noting that some antimony(III) hexahalogenides demonstrate semiconductor properties.^42^ However, this feature might be rather expected in the case of polymeric RMX_4_ and R_2_MX_5_ rather than for monomeric compounds. The well-known Kubelka–Munk relations were used to determine the energy band gap (Figure S15). The obtained value of ∼2.95 eV may suggest that the compound being studied does not meet the requirement to be classified as a semiconductor.

In summary, we have reported the first example of a hybrid haloantimonate(III) employing 1,2,4-triazolium as an aromatic cation displaying ferroelectric behavior, (C_2_N_3_H_4_)_3_[SbBr_6_] (**TBA**). The title compound undergoes two reversible PTs between tetragonal [*P*4_2_/*m* (I)] and monoclinic [*P*2_1_/*n* (II) and *P*2_1_ (III)] phases. **TBA** experiences a ferroelectric PT at 271/268 K (II–III) driven by “order–disorder” and “displacive” molecular mechanisms, whose ferroelectricity was confirmed by *P–E* hysteresis loop measurements. This finding provides a potential avenue for discrete (0D) haloantimonates(III) and halobismuthates(III) of the R_3_MX_6_ type to be used in optoelectronic applications.

## Supporting Information Available

Experimental description (Section 1); X-ray diffraction pattern at 298 K of **TBA** (red) and that calculated from the crystal structure (blue) (Figure S1, Section 2); thermal properties (Section 3), including simultaneous thermogravimetric (TGA) and differential thermal analysis (DTA) scans (ramp rate of 5 K min^–1^) (Figure S2) and DSC traces for **TBA** during the cooling and heating scans (rate of 5 K min^–1^, sample mass of 13.1430 mg) (Figure S3); crystal structure analysis (Section 4), including crystal data and abbreviated information about structure refinement results and periodic *ab initio*-calculated data for **TBA** (Table S1), the geometry of [SbBr_6_]^3–^ units (a, X-ray; b, calculated) in bonds and angles (Table S2), the geometry of hydrogen bonds in **TBA** at 100 K (a, X-ray; b, calculated) in bonds and angles (Table S3), **TBA** packing in the tetragonal phase [crystallographically unrelated cations A and B are distinguished by blue (A) and red (B) colors] (Figure S4), symmetry and orientation of the unit cell of the tetragonal, monoclinic centrosymmetric and monoclinic polar phases of **TBA** (Figure S5), comparison of **TBA** packing at 320 (left), 293 (center) and 100 K (right) (Figure S6), the scheme of the short (green) and long (orange) Sb–Br bonds in **TBA** at 293 and 100 K and the orientation of the dipole moment of the cations at 100 K with anions presented as octahedra (Figure S7) {[Sb(3)Br_6_]^3–^ octahedra colored green}, and N–H···Br hydrogen bond pattern in **TBA** at 100 K and π···π stacking interactions between cations from group B (−G–B–F–I–...) and the N–H···N and C–H···N hydrogen bonds in **TBA** at 100 K (Figure S8); SHG, including experimental SHG spectra collected during cooling and heating runs (Figure S9, Section 5); dielectric properties (Section 6), including the temperature dependence of the complex dielectric permittivity during a heating cycle (pellet sample) (Figure S10), the frequency dependence of the real and imaginary parts of permittivity at several temperatures (Figure S11), the dependence of ε″ versus ε′ for the single crystal of the **TBA** complex (Figure S12) (solid line is a fit to the Cole–Cole equation), and the temperature dependence of the macroscopic relaxation time (τ) and the microscopic relaxation time (τ_0_) and its inverse (τ_0_^–1^) above *T*_c_ (Figure S13); and UV–vis data (Section 7), including the UV–vis absorption spectrum of **TBA** in grease (Figure S14) and reflectance and absorption spectra of **TBA** (Figure S15) (PDF)
