# Supplementary material for: Progressive Structural Complexity in Ferroelectric 1,2,4-Triazolium Hexabromoantimonate(III): Interplay of “Order–Disorder” and “Displacive” Contributions to the Structural Phase Transitions
Source: J Phys Chem Lett. 2023 May 9;14(19):4524–31. doi: 10.1021/acs.jpclett.3c00924 (PMC10201570; doi:10.1021/acs.jpclett.3c00924)
Supplement: Supplementary file 1 — jz3c00924_si_001.pdf [file jz3c00924_si_001.pdf]

SUPPLEMENTARY INFORMATION

Progressive Structural Complexity in Ferroelectric 1,2,4-Triazolium Hexabromoantimonate(III): Interplay of 'Order-Disorder' and 'Displacive' Contributions to the Structural Phase Transitions

*Michał Chański, Agata Białońska, Ryszard Jakubas, Magdalena Rok, Jan K. Zaręba[†], Rafał Janicki, Piotr Durlak and A. Piecha-Bisiorek**

Faculty of Chemistry, University of Wrocław,50-383, Poland

[†] Institute of Advanced Materials, Faculty of Chemistry, Wrocław University of Science and Technology, Wrocław 50-370, Poland

Corresponding Author: anna.piecha-bisiorek@uwr.edu.pl (Anna Piecha-Bisiorek)

Table of contents:

Section 1: Experimental description
Section 2: PXRD
Figure S1. The X-ray diffraction pattern at 298 K of **TBA** (red) and calculated from crystal structure (blue).
Section 3: Thermal properties
Figure S2. Simultaneous thermogravimetric (TGA) and differential thermal analyses (DTA) scan (ramp rate: 5 K min^{-1}).
Figure S3. DSC traces for **TBA** during the cooling and heating scans (rate: 5 K·min^{-1}, sample mass 13.1430 mg).
Section 4: Crystal structure analysis
Table S1. Crystal data and abbreviated information about structure refinement results and periodic *ab initio* calculated data for **TBA**.
Table S2. The geometry of $[SbBr_6]^{3-}$ units (^{a} - X-ray, ^{b} - calc.) bonds in [Å]; angles in [deg]
Table S3. The geometry of hydrogen bonds in **TBA** at 100 K (^{a} - X-ray, ^{b} - calc.) bonds in [Å]; angles in [deg]
Figure S4. The **TBA** packing in the tetragonal phase (crystallographically unrelated cations A and B are distinguished by blue (A) and red (B) color).
Figure S5. The symmetry and the orientation of the unit cell of the tetragonal, monoclinic centrosymmetric and monoclinic polar phases of **TBA**.
Figure S6. Comparison of **TBA** packing at 320 (left), 293 (center) and 100 K (right).
Figure S7. The scheme of the short (green) and the long (orange) Sb-Br bond in **TBA** at (a) 293 K (b) 100 K and the orientation of the dipole moment of the cations (c) at 100 K with anions presented as octahedra. The $[Sb(3)Br_6]^{3-}$ octahedra are distinguished by green color.
Figure S8. (a) The N-H…Br hydrogen bonds pattern in **TBA** at 100 K; (b) The π…π stacking interactions between cations from group B (-G-B-F-I-…) and the N-H…N and C-H…N hydrogen bonds in **TBA** at 100 K.
Section 5: SHG
Figure S9. Experimental SHG spectra collected during a) cooling and b) heating runs.
Section 6: Dielectric properties
Figure S10. Temperature dependence of the complex dielectric permittivity during heating cycle (pellet sample).
Figure S11. The frequency dependence of (a) the real and (b) the imaginary part of permittivity at several temperatures.
Figure S12. The dependence of ε'' versus ε' for the single crystal of the **TBA** complex. The solid line represents fit to the Cole–Cole equation.
Figure S13. Temperature dependence of the macroscopic (τ) and microscopic (τ_0) relaxation time and its inverse (τ_0^{-1}) above *T*c.
Section 7: UV-vis
Figure S14. UV-vis absorption spectrum of TBA in grease.
Figure S15. Reflectance (a) and absorption (b) spectra of **TBA**.

SECTION 1: Experimental

1.1 Sample preparation

All the materials needed for the synthesis of $(C_2N_3H_4)_3[SbBr_6]$ (**TBA**) were purchased from commercial sources (Sigma-Aldrich) and used without further purification: 1,2,4-triazole (98%), Sb_2O_3 (>99.998%), and HBr (48%). The crystals were grown by the slow evaporation of a concentrated HBr solution containing 3:1 ratio of $C_2H_3N_3$ and Sb_2O_3. The salts obtained were recrystallized twice from a methanol solution, and their compositions were verified by elemental analysis: C: 8.72% (*theor*. 8.88%), N: 15.66% (*theor*. 15.54%), and H: 1.62% (*theor*. 1.49%). The single crystals were grown from an aqueous solution at room temperature (RT).

1.2 Thermal analysis

DSC measurements were performed by heating and cooling of the polycrystalline sample in the temperature range of 100–320 K with a ramp rate of 5 $K \cdot min^{-1}$ using a Metler Toledo DSC 3 instrument. The TGA/DSC measurements were performed on a TGA-DSC 3+ instrument between 290 and 740 K with a ramp rate of 5 K min^{-1}. The scan was performed in flowing nitrogen (flow rate: 1 dm^3 h^{-1}).

1.3 Crystal structure determination

The X-ray data of **TBA** were collected at 320 K, 293 K and 100 K using an Oxford Cryosystem device. X-ray data were collected on a Xcalibur Sapphire2 diffractometer (MoKα radiation; λ = 0.71073 Å). Data reduction and analysis were carried out with the CrysAlis 'RED' program.[1] Space groups were determined, based on systematic absences and intensity statistics. Structures were solved by Patterson method using the SHELXS program and refined using all F^2 data, as implemented by the SHELXL programs.[2] Positions of carbon and nitrogen atoms in triazolium cations were chosen on the base of distances to potential Br and N acceptors of hydrogen bonds. In **TBA** at 293 K and at 320 K most of triazolium cations are disordered and the C/N arrangement in the disordered triazolium rings is unreliable. Non-hydrogen atoms were refined with anisotropic displacement parameters. However, in the structure at 293 K and at 320 K, the geometry of the disordered triazole rings and displacement parameters of their atoms were restrained using AFIX and ISOR commands, respectively. Moreover, for partially overlapping atoms, SIMU restrain was applied. At 293 K, occupancy factor of the disordered triazole rings were refined assuming that the sum of all components of a given disordered cation located in general and in the special positions is equal to 1 and 0.5, respectively. In the crystal structure at 293 K and at 320 K, H atoms were not found. At 100 K, all H atoms were found in $\Delta\rho$ map, and before the last refinement cycle, they were fixed and were allowed to ride on their parent atoms.

1.4 Electric properties

Electrical measurements of TBA were performed on polycrystalline samples in the form of pressed pellets with geometrical parameters (S = 20–25 mm^2, d = 0.8–0.4 mm). The complex dielectric permittivity was measured between 250 and 320 K by an Agilent E4980A Precision LCR Meter in the frequency range of 135 Hz–2 MHz. The electric measurements were carried out in a controlled nitrogen atmosphere. The overall error for the complex permittivity real and imaginary parts was less than 5%. The ferroelectric hysteresis loops were obtained by using a Sawyer–Tower circuit Precision Premier II (Radiant Technologies, Inc.) at a frequency of 50 Hz. The surfaces of the pellet were coated with a gold electrode with a mask using a sputter coating system (Quorum Q150T S).

1.5. Second harmonic generation (SHG)

Temperature-resolved SHG studies were performed using a laser system employing a wavelength-tunable Topaz Prime Vis-NIR optical parametric amplifier (OPA) pumped by Coherent Astrella Ti: Sapphire regenerative amplifier providing femtosecond laser pulses (800 nm, 75 fs) at 1 kHz repetition rate. The output of OPA was set to 1300 nm and was used unfocused. Laser fluence at samples was equal to 0.25 mJ/cm^2.

The single crystals of TBA were crushed with a spatula and sieved through an Aldrich mini-sieve set, collecting a microcrystal size fraction of 125–177 μm. Next, size-graded samples were fixed in-between microscope glass slides to form tightly packed layers, sealed, and mounted to the horizontally aligned

sample holder. No refractive index matching oil was used. The employed measurement setup operates in the reflection mode. Specifically, the laser beam delivered from OPA was directed onto the sample at 45 degrees to its surface. Emission collecting optics consisted of a Ø25.0 mm plano-convex lens of focal length 25.4 mm mounted to the 400 µm 0.22 NA glass optical fiber and was placed along the normal to the sample surface. The distance between the collection lens and the sample was equal to 30 mm. The spectra of the nonlinear optical responses were recorded by an Ocean Optics Flame T fiber-coupled CCD spectrograph with a 200 µm entrance slit. Scattered pumping radiation was suppressed with the use of a Thorlabs 750 nm short-pass dielectric filter (FESH0750). Temperature control of the sample was performed using a Linkam LTS420 Heating/Freezing Stage. Temperature stability was equal to 0.1 K.

1.6. Computational Methods (Periodic Ab Initio Calculations)

Quantum-mechanical condensed matter simulations including: a series of full geometry and cell parameters optimizations, spontaneous polarisation (P_s), electronic band structure (EBS) of crystal, the band gap, and density of states (DOS) calculations were performed to localize the key stationary points on the potential energy surface (PES) of the **TBA**. These calculations employed the London-type empirical correction in the (D3) variant for dispersion interactions as proposed by Grimme[3–6] including three-body dispersion contributions with fast analytical gradients together with the vibrational harmonic frequency calculations. The structural data (starting geometry) were taken from the X-ray crystal structure of TBA from this present study. Calculations were performed using the CRYSTAL17 software,[7,8] utilizing the DFT-D3[3–6] methods with the hybrid functional: the Becke's three-parameter functional combined with the nonlocal correlation Lee–Yang–Parr (B3LYP-D3)[9–11] with the two shrinking factors (4',4') to generate a commensurate grid of k-points in reciprocal space, following the Monkhorst–Pack[12] net method. All calculations were carried out with the consistent gaussian basis sets of double zeta valence with polarization quality for solid-state calculations (pob_DZVP_rev2) in second revision include BSSE-correction scheme as proposed by Peintinger, Vilela Oliveira, Laun and Bredow[13–16]. For the antimony atom we have used for the calculation Sb_pob_DZVP_2018 basis set[15] which is an extension of the pob_DZVP basis set, and is based on the full-relativistic effective core potentials (ECPs) of the Stuttgart/Cologne group and on the def2-SVP valence basis[16,17] of the Ahlrichs group. To check if the crystal structure of TBA is at the global minimum on the PES after optimization, IR harmonic frequencies were calculated. The imaginary frequencies were not found. For the **TBA** crystal, vibrational frequency calculations using CRYSTAL17 were performed at the Γ-point[18,19]. The spontaneous polarisation (P_s) in the TBA crystal was calculated and evaluated through either a Berry phase (BP) approach[20–22] as the polarization difference between one of the two enantiomorphic structures (λ=+1 or λ=−1) and the intermediate geometric structure (λ=0). The electronic band structure was generated according to the procedure in the CRYSTAL17 program. In order to prepare the input file, the SeeK-path[23] tool was also used, and the EBS and DOS were visualized in CRYSPLOT [24] and Gnuplot [25] programs.

1.7. UV-vis spectroscopy

The diffuse reflectance and absorption UV-vis spectra at room temperature were recorded with Cary 5000 spectrophotometer.

SECTION 2: PXRD

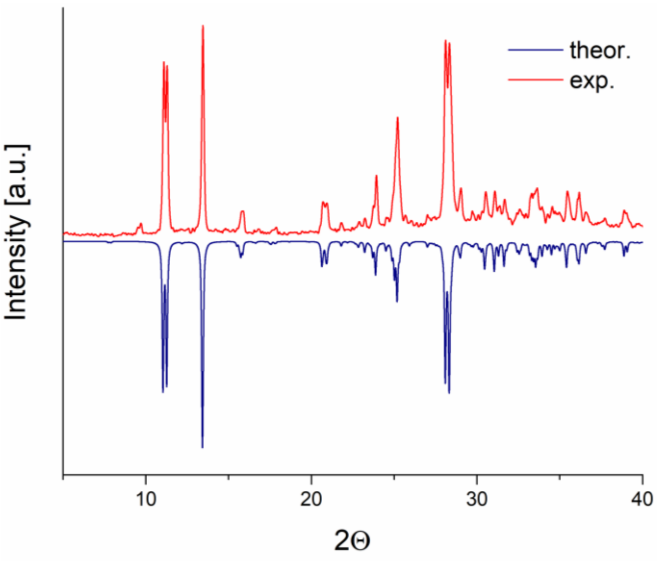

Figure S1 The X-ray diffraction pattern at 298 K of **TBA** (red) and calculated from crystal structure (blue).

SECTION 3: Thermal properties

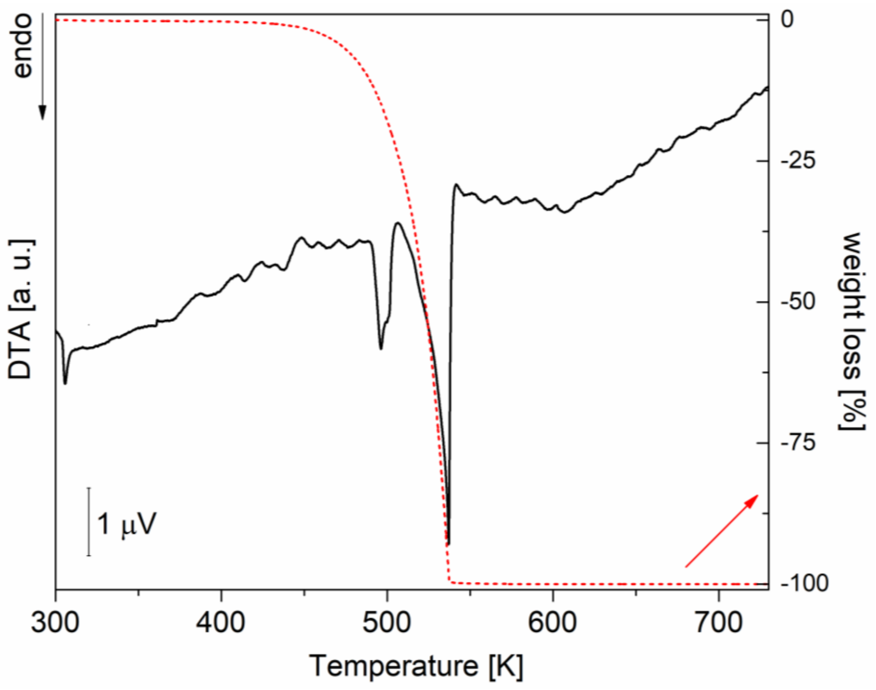

Figure S2. Simultaneous thermogravimetric (TGA) and differential thermal analyses (DTA) scan (ramp rate: 5 K min^{-1}).

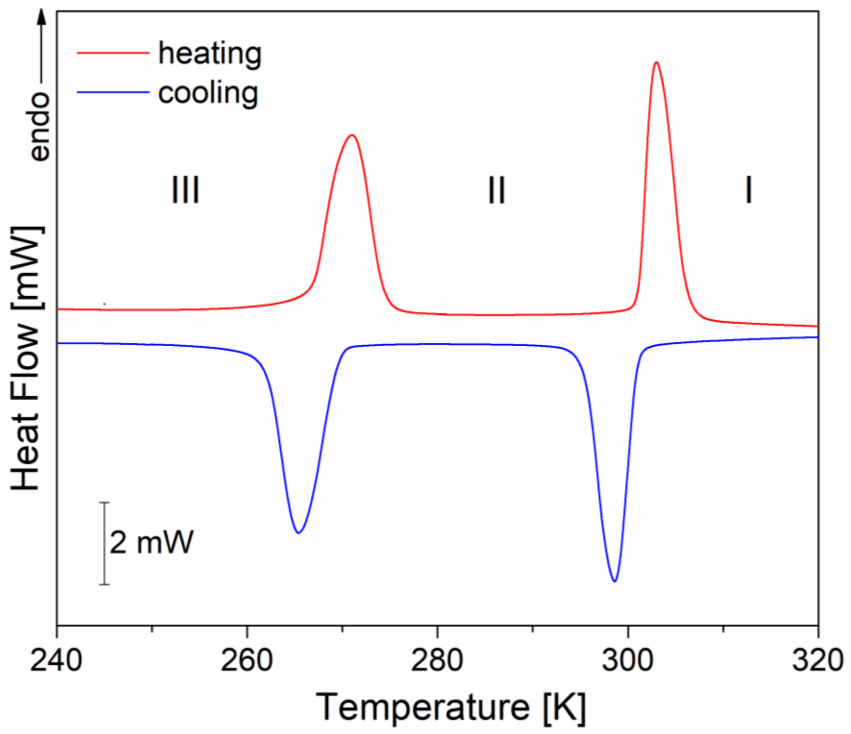

Figure S3. DSC traces for **TBA** during the cooling and heating scans (rate: 5 K·min^{-1}, sample mass 13.1430 mg).

SECTION 4: Crystal structure analysis

Table S1. Crystal data and abbreviated information about structure refinement results and periodic *ab initio* calculated data for **TBA**.

Phase	**I**	**II**	**III**	**III (calc.)**
Chemical formula	$(C_2H_4N_3)_3[SbBr_6]$	$(C_2H_4N_3)_3[SbBr_6]$	$(C_2H_4N_3)_3[SbBr_6]$	$(C_2H_4N_3)_3[SbBr_6]$
M_R	811.46	811.46	811.46	-
Temperature (K)	320	293	100	0
Crystal system, space group	tetragonal $P4_2/m$	monoclinic $P2_1/n$	monoclinic $P2_1$	monoclinic $P2_1$
a, b, c (Å)	11.149(3)<br>11.149(3)<br>8.318(2)	15.699(3)<br>16.294(3)<br>16.018(3)	15.361(3)<br>16.236(3)<br>15.858(3)	14.9323<br>15.5281<br>15.1215
β (°)	90	90.41(3)	90.49(3)	88.87
V (Å^3)	1033.9(6)	4097.3(13)	3954.9(13)	3505.5
Z	2	8	8	8
D_c (Mg m^{-3})	2.606	2.631	2.726	-
$R[F^2>2s(F^2)]$, $wR(F^2)$, S	0.073, 0.246, 0.763	0.106, 0.201, 1.159	0.043, 0.073, 1.072	-
**CCDC number**	**2253552**	**2253553**	**2253554**	-

Table S2. The geometry of $[SbBr_6]^{3-}$ units ([a] - X-ray, [b] - calc.) bonds in [Å]; angles in [deg]

320 K	Sb1 Br1 2.798(2)<br>Sb1 Br3 2.7995(16)<br>Br1 Sb1 Br3 92.89(5)			
293 K	Sb1 Br1 2.660(2)<br>Sb1 Br5 2.787(2)<br>Sb1 Br3 2.788(2)<br>Sb1 Br4 2.813(2)<br>Sb1 Br6 2.847(2)<br>Sb1 Br2 2.975(2)<br>Br1 Sb1 Br5 93.56(8)<br>Br1 Sb1 Br3 88.56(8)<br>Br5 Sb1 Br3 91.10(7)<br>Br1 Sb1 Br4 93.67(8)<br>Br5 Sb1 Br4 92.06(7)<br>Br3 Sb1 Br4 176.01(8)<br>Br1 Sb1 Br6 88.02(8)<br>Br5 Sb1 Br6 178.19(8)<br>Br3 Sb1 Br6 88.07(7)<br>Br4 Sb1 Br6 88.70(7)<br>Br1 Sb1 Br2 178.16(9)<br>Br5 Sb1 Br2 87.92(7)	Sb2 Br7 2.730(2)<br>Sb2 Br11 2.756(2)<br>Sb2 Br9 2.757(2)<br>Sb2 Br12 2.857(2)<br>Sb2 Br10 2.864(2)<br>Sb2 Br8 2.945(3)<br>Br7 Sb2 Br11 89.17(8)<br>Br7 Sb2 Br9 90.12(7)<br>Br11 Sb2 Br9 91.51(7)<br>Br7 Sb2 Br12 89.89(7)<br>Br11 Sb2 Br12 178.91(8)<br>Br9 Sb2 Br12 89.04(7)<br>Br7 Sb2 Br10 87.84(7)<br>Br11 Sb2 Br10 88.94(7)<br>Br9 Sb2 Br10 177.90(8)<br>Br12 Sb2 Br10 90.48(7)<br>Br7 Sb2 Br8 172.01(8)<br>Br11 Sb2 Br8 96.60(8)		

	Br3 Sb1 Br2 90.34(7)	Br9 Sb2 Br8 84.24(7)		
	Br4 Sb1 Br2 87.35(7)	Br12 Sb2 Br8 84.39(7)		
	Br6 Sb1 Br2 90.48(7)	Br10 Sb2 Br8 97.75(8)		
100 K	Sb1 Br1 2.6329(13)[a] 2.71[b]	Sb2 Br8 2.6526(13)[a] 2.72[b]	Sb3 Br14 2.6381(13)[a] 2.72[b]	Sb4 Br20 2.6445(13)[a] 2.71[b]
	Sb1 Br5 2.7099(11)[a] 2.71[b]	Sb2 Br10 2.7106(11)[a] 2.76[b]	Sb3 Br16 2.6674(11)[a] 2.76[b]	Sb4 Br22 2.7579(12)[a] 2.71[b]
	Sb1 Br4 2.7383(11)[a] 2.72[b]	Sb2 Br11 2.7908(11)[a] 2.72[b]	Sb3 Br18 2.7620(12)[a] 2.72[b]	Sb4 Br23 2.7876(11)[a] 2.72[b]
	Sb1 Br3 2.8792(11)[a] 2.72[b]	Sb2 Br12 2.8249(11)[a] 2.72[b]	Sb3 Br17 2.8055(11)[a] 2.72[b]	Sb4 Br24 2.8003(12)[a] 2.72[b]
	Sb1 Br6 2.9044(12)[a] 2.76[b]	Sb2 Br9 2.8815(12)[a] 2.76[b]	Sb3 Br13 2.9957(13)[a] 2.76[b]	Sb4 Br21 2.8574(12)[a] 2.76[b]
	Sb1 Br2 3.0008(13)[a] 2.77[b]	Sb2 Br7 3.0612(13)[a] 2.78[b]	Sb3 Br15 3.0525(12)[a] 2.78[b]	Sb4 Br19 3.0821(14)[a] 2.77[b]
	Br1 Sb1 Br5 91.25(4)[a] 94.4[b]	Br8 Sb2 Br10 89.40(4)[a] 94.4[b]	Br14 Sb3 Br16 88.49(4)[a] 94.4[b]	Br20 Sb4 Br22 90.53(4)[a] 94.4[b]
	Br1 Sb1 Br4 92.04(4)[a] 95.6[b]	Br8 Sb2 Br11 88.34(4)[a] 90.2[b]	Br14 Sb3 Br18 94.00(4)[a] 96.3[b]	Br20 Sb4 Br23 87.83(4)[a] 90.4[b]
	Br5 Sb1 Br4 93.52(4)[a] 96.3[b]	Br10 Sb2 Br11 89.63(4)[a] 96.3[b]	Br16 Sb3 Br18 89.91(4)[a] 90.2[b]	Br22 Sb4 Br23 87.21(4)[a] 90.2[b]
	Br1 Sb1 Br3 90.11(4)[a] 89.3[b]	Br8 Sb2 Br12 87.29(4)[a] 89.3[b]	Br14 Sb3 Br17 89.15(4)[a] 89.3[b]	Br20 Sb4 Br24 87.22(4)[a] 89.3[b]
	Br5 Sb1 Br3 89.89(4)[a] 85.2[b]	Br10 Sb2 Br12 89.95(4)[a] 85.2[b]	Br16 Sb3 Br17 88.00(4)[a] 85.2[b]	Br22 Sb4 Br24 92.12(4)[a] 96.3[b]
	Br4 Sb1 Br3 175.93(4)[a] 179.5[b]	Br11 Sb2 Br12 175.62(4)[a] 179.5[b]	Br18 Sb3 Br17 176.18(4)[a] 179.5[b]	Br23 Sb4 Br24 175.00(4)[a] 179.5[b]
	Br1 Sb1 Br6 86.74(4)[a] 80.5[b]	Br8 Sb2 Br9 86.23(4)[a] 80.5[b]	Br14 Sb3 Br13 173.42(4)[a] 178.4[b]	Br20 Sb4 Br21 88.80(4)[a] 85.2[b]
	Br5 Sb1 Br6 175.45(4)[a] 178.4[b]	Br10 Sb2 Br9 175.36(4)[a] 178.4[b]	Br16 Sb3 Br13 85.21(4)[a] 80.5[b]	Br22 Sb4 Br21 176.88(4)[a] 178.4[b]
	Br4 Sb1 Br6 90.64(4)[a] 89.3[b]	Br11 Sb2 Br9 88.68(4)[a] 85.2[b]	Br18 Sb3 Br13 87.91(4)[a] 85.2[b]	Br23 Sb4 Br21 89.72(4)[a] 85.2[b]
	Br3 Sb1 Br6 86.04(3)[a] 80.5[b]	Br12 Sb2 Br9 91.40(4)[a] 89.3[b]	Br17 Sb3 Br13 88.72(4)[a] 85.2[b]	Br24 Sb4 Br21 90.89(4)[a] 89.3[b]
	Br1 Sb1 Br2 174.80(4)[a] 178.4[b]	Br7 Sb2 Br8 166.90(4)[a] 168.4[b]	Br15 Sb3 Br13 90.03(3)[a] 89.3[b]	Br19 Sb4 Br20 169.28(4)[a] 168.4[b]
	Br5 Sb1 Br2 85.43(4)[a] 80.5[b]	Br7 Sb2 Br9 100.49(4)[a] 103.6[b]	Br15 Sb3 Br14 96.17(4)[a] 96.3[b]	Br19 Sb4 Br21 99.69(4)[a] 96.3[b]
	Br4 Sb1 Br2 84.19(3)[a] 80.5[b]	Br7 Sb2 Br10 83.49(4)[a] 80.5[b]	Br15 Sb3 Br16 174.55(4)[a] 178.4[b]	Br19 Sb4 Br22 80.63(4)[a] 80.5[b]
	Br3 Sb1 Br2 93.87(4)[a] 94.4[b]	Br7 Sb2 Br11 80.66(3)[a] 80.5[b]	Br15 Sb3 Br17 89.23(3)[a] 85.2[b]	Br19 Sb4 Br23 85.70(4)[a] 80.5[b]
	Br6 Sb1 Br2 96.85(4)[a] 96.3[b]	Br7 Sb2 Br12 103.63(4)[a] 103.7[b]	Br15 Sb3 Br18 92.58(4)[a] 94.4[b]	Br19 Sb4 Br24 99.08(4)[a] 96.3[b]

Table S3. The geometry of hydrogen bonds in **TBA** at 100 K ([a] - X-ray, [b] - calc.) bonds in [Å]; angles in [deg]

	H…A		D…A		∡DHA	
	X-ray	Calc.	X-ray	Calc.	X-ray	Calc.
N2A-H2A...Br13_$1	2.59	2.58	3.294(9)	3.28	140	139.5
N4A-H4A...N2F	2.18	2.19	3.027(12)	3.02	170	170.3
N1B-H1B...Br19	2.79	2.77	3.461(8)	3.48	136	137.2
N4B-H4B...Br9	2.50	2.51	3.312(8)	3.32	157	158.1
N2C-H2C...Br7	2.64	2.65	3.322(8)	3.32	137	134.6
N4C-H4C...N2B_$1	2.07	2.03	2.915(11)	2.90	168	165.3
N2D-H2D...N1C	2.04	2.03	2.841(11)	2.83	155	155.2
N4D-H4D...Br3_$3	2.49	2.47	3.329(8)	3.33	167	165.6
C3E-H3E...N2G	2.47	2.46	3.279(14)	3.25	146	146.2
N4E-H4E...Br24_$5	2.63	2.65	3.462(10)	3.46	164	168.4
N1F-H1F...Br6	2.59	2.57	3.335(9)	3.34	145	144.7
N4F-H4F...Br17_$6	2.89	2.91	3.568(9)	3.62	137	132.3
N4F-H4F...N2H_$7	2.26	2.25	2.939(12)	2.92	136	139.5
N1G-H1G...Br4	2.70	2.69	3.339(8)	3.34	132	133.4

N1G-H1G...Br2	2.81	2.83	3.504(8)	3.52	140	138.3
N4G-H4G...Br15	2.43	2.44	3.261(8)	3.28	164	168.2
N1H-H1H...Br13_$1	2.60	2.58	3.359(8)	2.37	148	144.7
N4H-H4H...Br2	2.69	2.70	3.400(8)	3.41	140	136.4
C5H-H5H...N1A	2.50	2.51	3.353(12)	3.37	153	150.3
N1I-H1I...Br7_$7	2.56	2.53	3.324(8)	3.34	149	153.4
N4I-H4I...Br21_$1	2.45	2.44	3.275(8)	3.30	162	158.2
N2J-H2J...Br19_$9	2.57	2.56	3.272(9)	3.30	139	130.5
N4J-H4J...N2I_$9	2.10	2.11	2.951(12)	2.89	170	176.3
N1K-H1K...N1J_$3	2.06	2.04	2.859(12)	2.84	155	150.2
N4K-H4K...Br15_$10	2.56	2.57	3.393(9)	3.36	162	163.2
N1L-H1L...N1E	2.10	2.11	2.864(12)	2.84	148	144.7
N4L-H4L...Br12_$7	2.64	2.62	3.441(8)	3.43	156	160.8

The symmetry codes: $1 x, y, z+1; $2 -x+2, y-1/2, -z; $3 -x+1, y-1/2, -z+1; $4 -x+2, y-1/2, -z+1; $5 -x+1, y+1/2, -z; $6 x-1, y, z+1; $7 x-1, y, z; $8 -x+2, y+1/2, -z+1; $9 -x+1, y+1/2, -z+1; $10 -x+1, y-1/2, -z

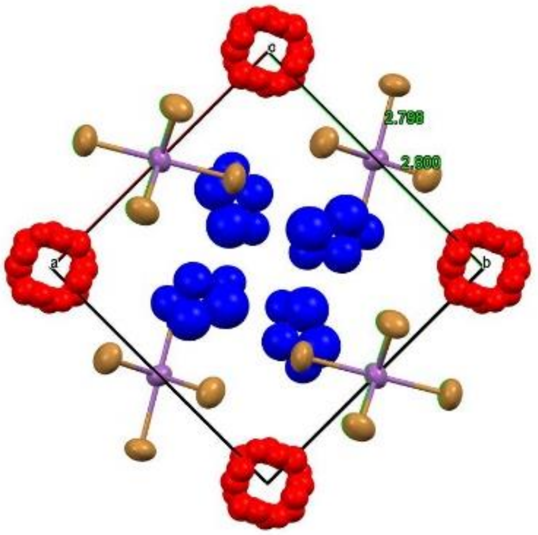

Figure S4. The **TBA** packing in the tetragonal phase (crystallographically unrelated cations A and B are distinguished by blue (A) and red (B) color).

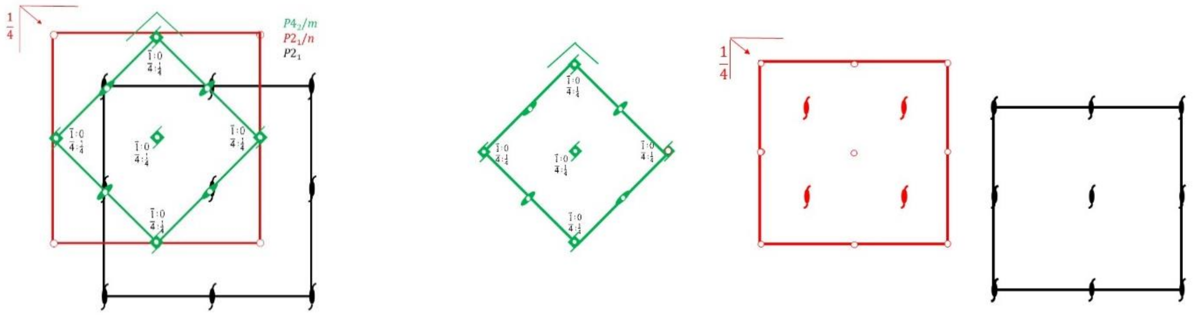

Figure S5. The symmetry and the orientation of the unit cell of the tetragonal, monoclinic centrosymmetric and monoclinic polar phases of **TBA**.

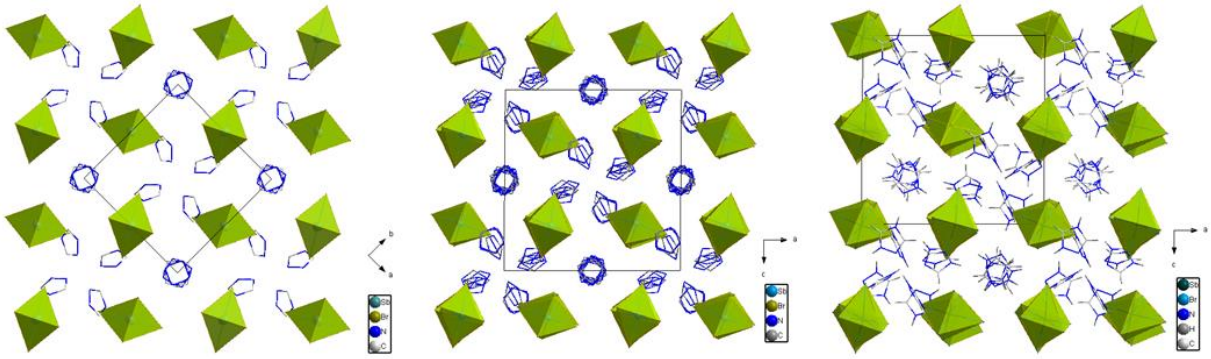

Figure S6. Comparison of **TBA** packing at 320 (left), 293 (center) and 100 K (right).

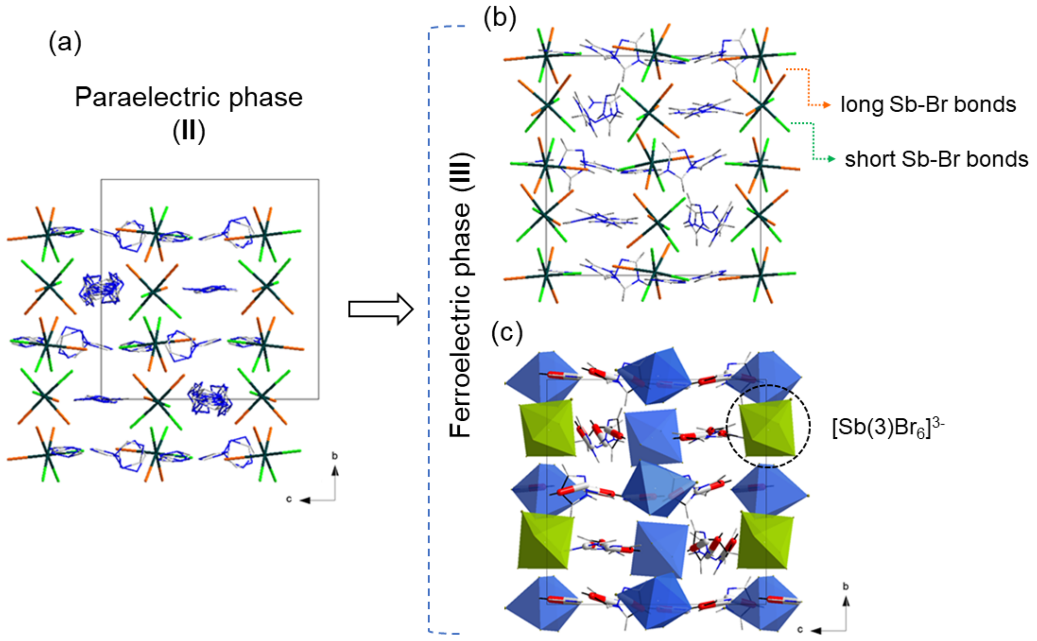

Figure S7. The scheme of the short (green) and the long (orange) Sb-Br bond in **TBA** at (a) 293 K (b) 100 K and the orientation of the dipole moment of the cations (c) at 100 K with anions presented as octahedra. The $[Sb(3)Br_6]^{3-}$ octahedra are distinguished by green color.

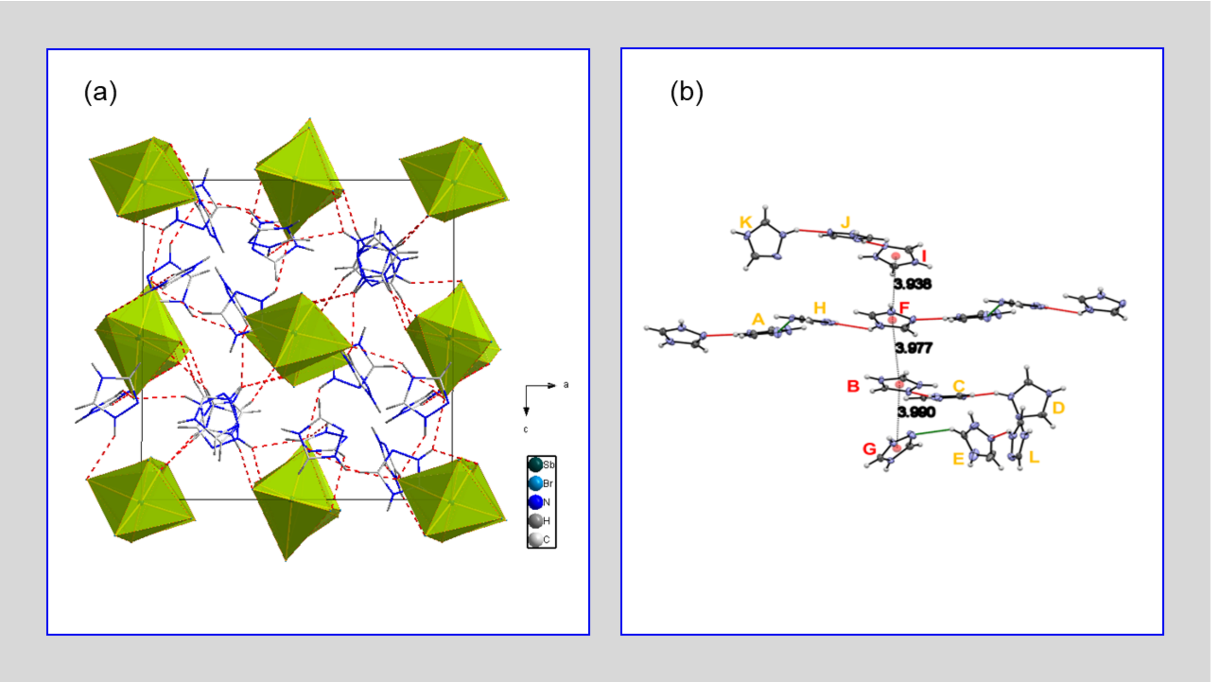

Figure S8. (a) The N-H…Br hydrogen bonds pattern in **TBA** at 100 K; (b) The π…π stacking interactions between cations from group B (-G-B-F-I-…) and the N-H…N and C-H…N hydrogen bonds in **TBA** at 100 K.

SECTION 5: SHG

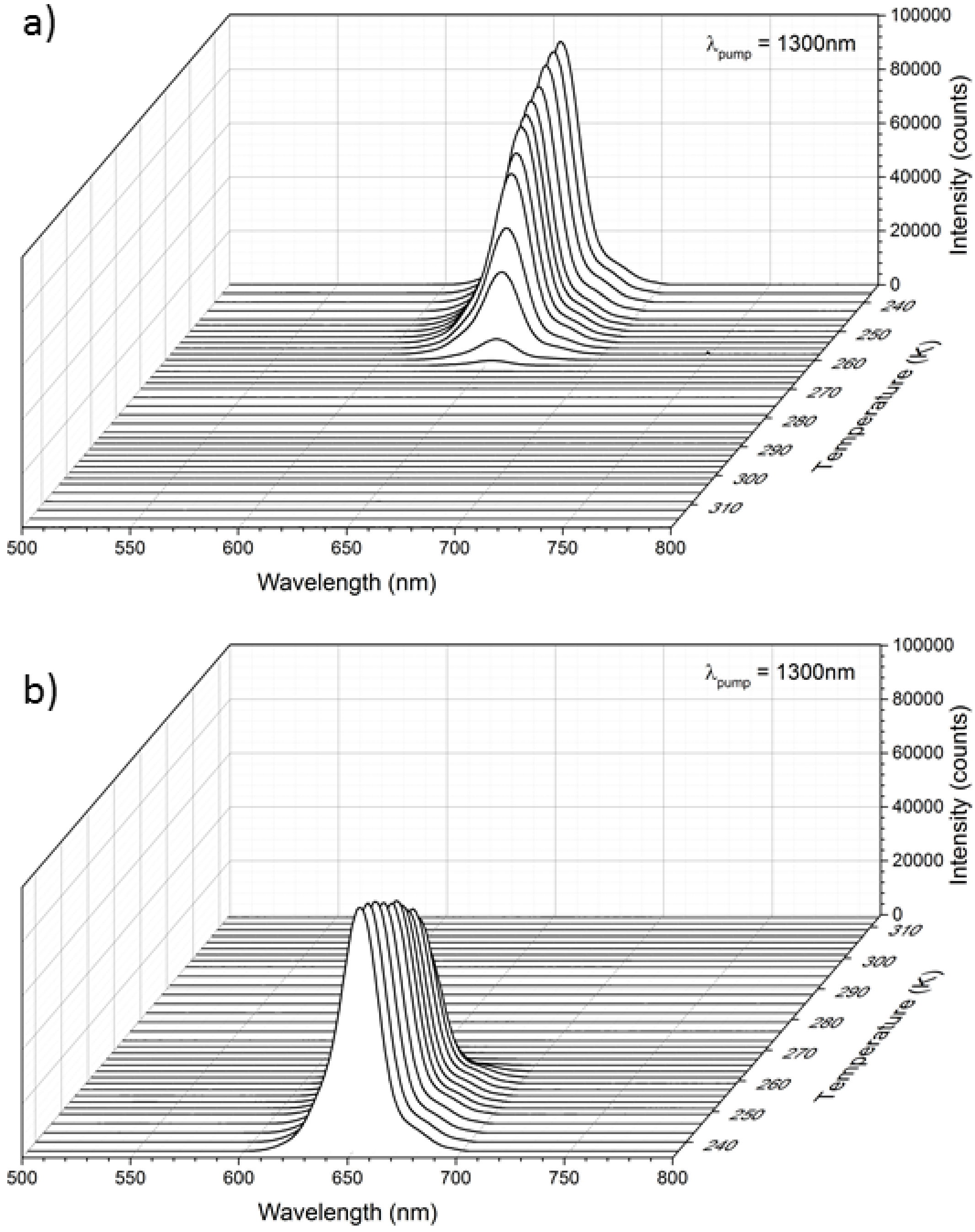

Figure S9. Experimental SHG spectra collected during a) cooling and b) heating runs.

SECTION 6: Dielectric properties

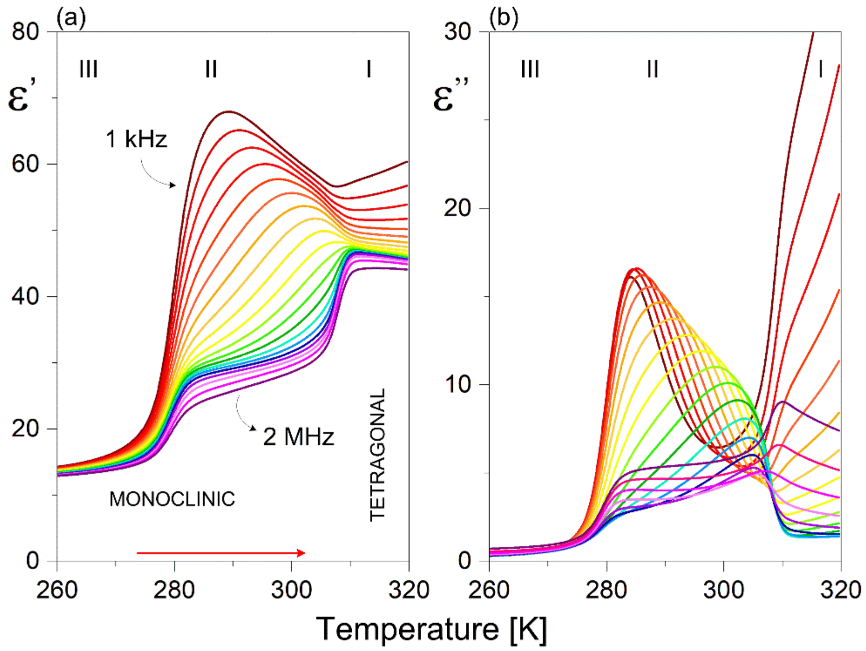

Figure S10. Temperature dependence of the complex dielectric permittivity during heating cycle (pellet sample).

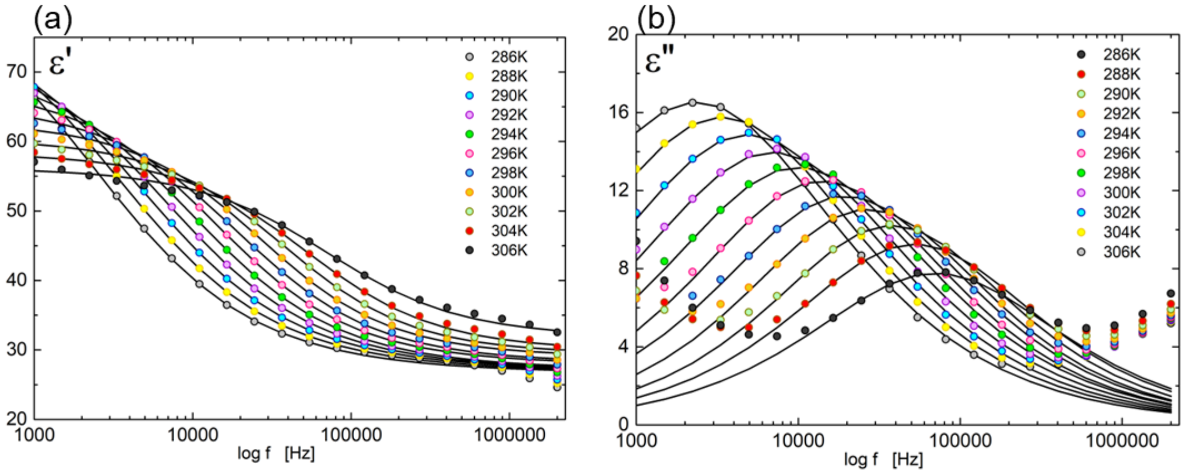

Figure S11. The frequency dependence of (a) the real and (b) the imaginary part of permittivity at several temperatures.

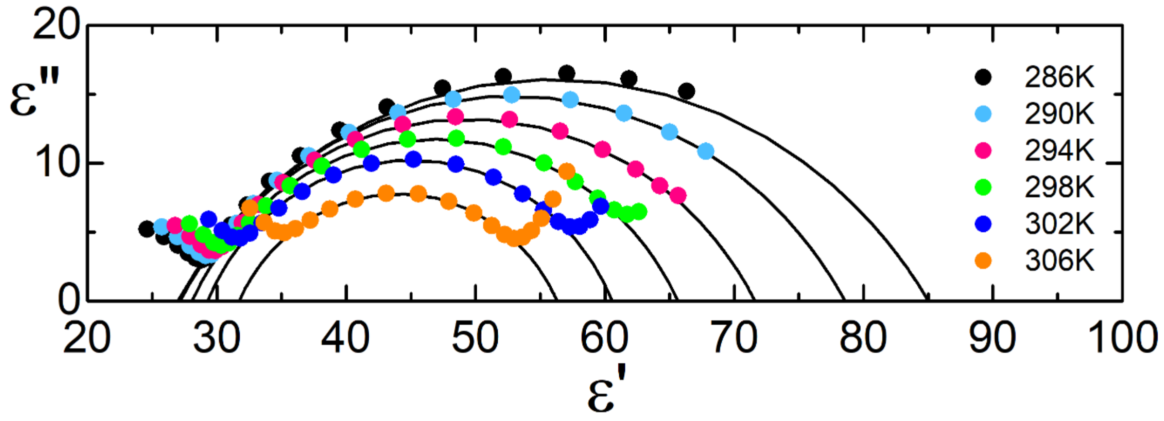

Figure S12. The dependence of ε" versus ε’ for the single crystal of the **TBA** complex. The solid line represents fit to the Cole–Cole equation.

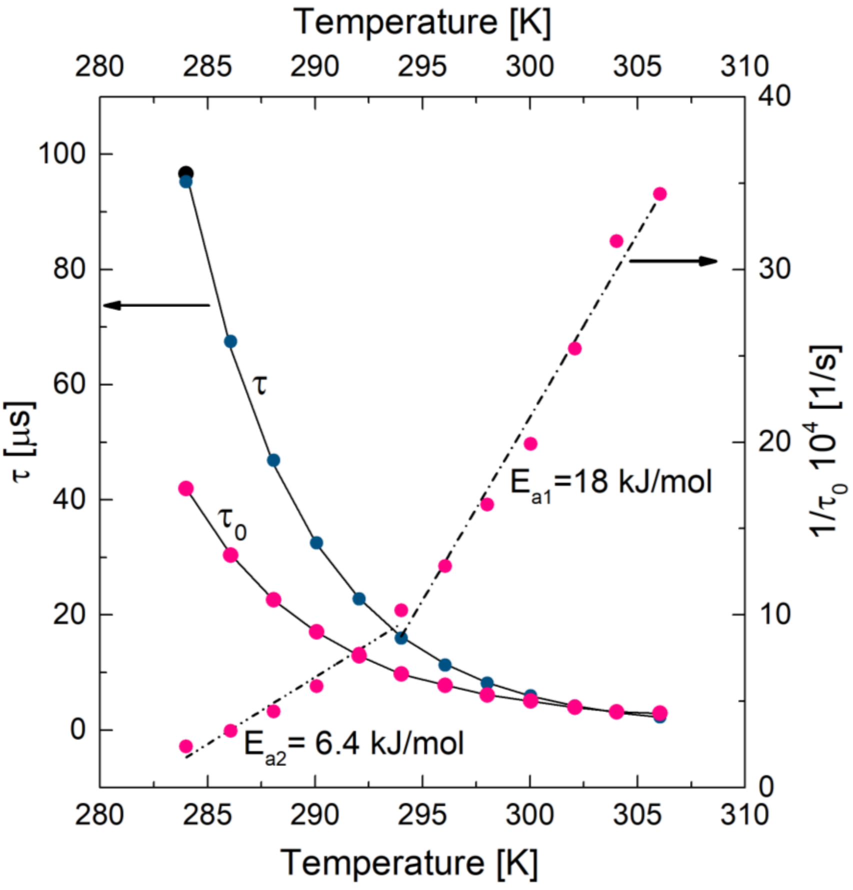

Figure S13. Temperature dependence of the macroscopic (τ) and microscopic (τ_0) relaxation time and its inverse (τ_0^{-1}) above Tc.

SECTION 7: UV-vis

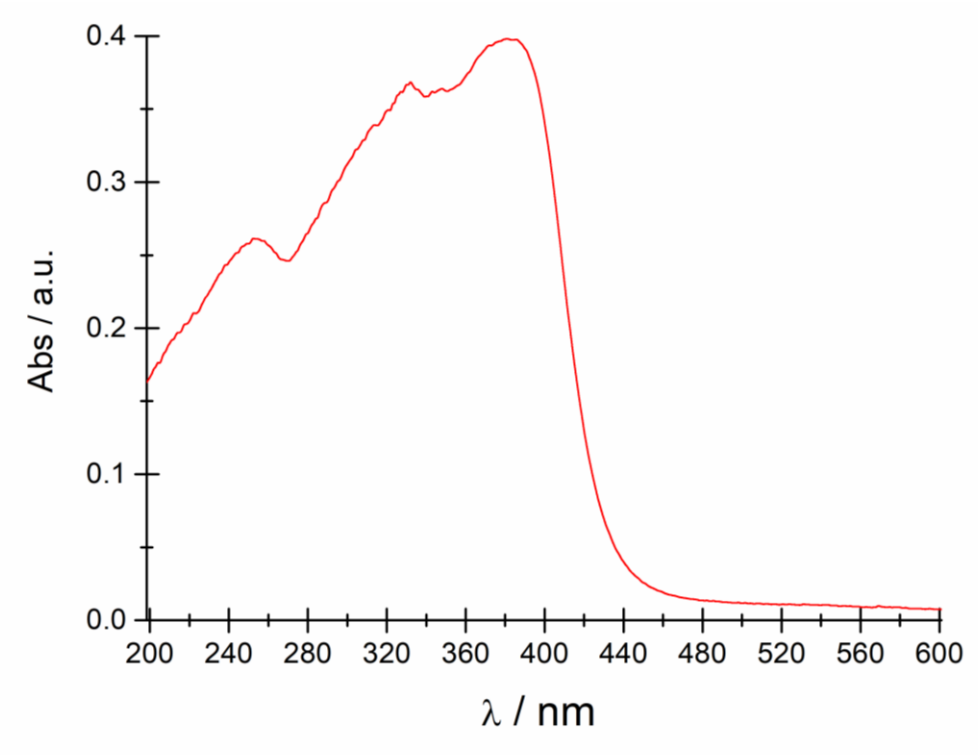

Figure S14. UV-vis absorption spectrum of TBA in grease.

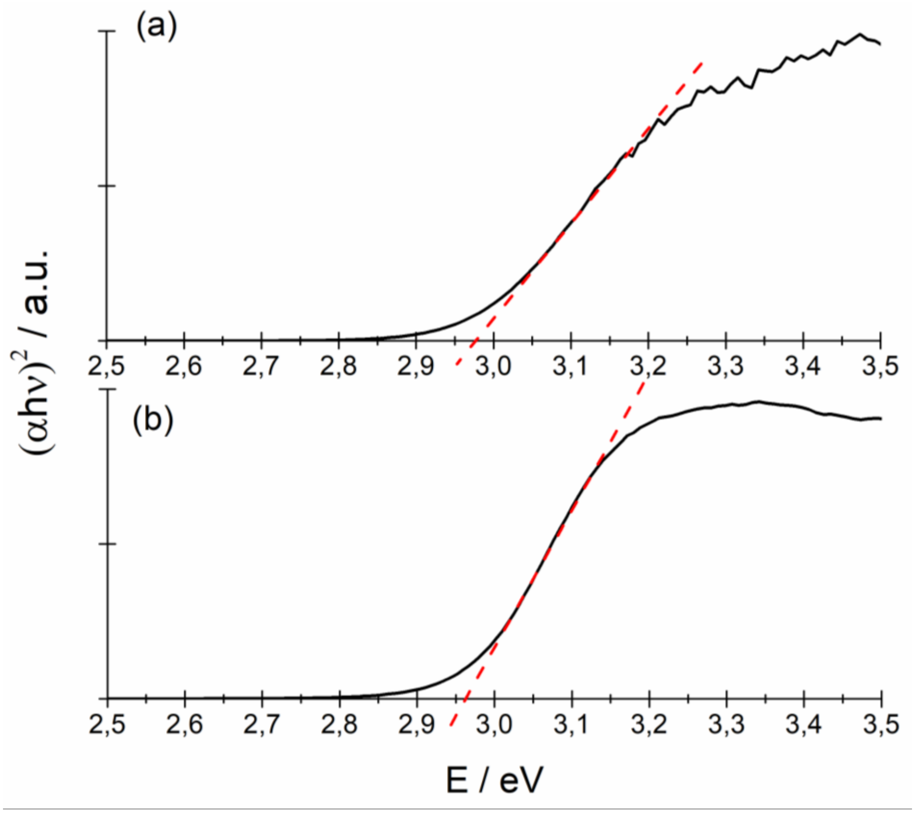

Figure S15. Reflectance (a) and absorption (b) spectra of **TBA**.

References:

(1) Sheldrick, G. M. A Short History of SHELX. *Acta Crystallogr. Sect. A Found. Crystallogr.* **2007**, *64*, 112–122.

(2) Sheldrick, G. M. Crystal Structure Refinement with SHELXL. *Acta Crystallogr. Sect. C Struct. Chem.* **2015**, *71* (Md), 3–8.

(3) Grimme, S. Semiempirical GGA-Type Density Functional Constructed with a Long-Range Dispersion Correction STEFAN. *J. Comput. Chem.* **2066**, *27*, 1787–1799.

(4) Grimme, S.; Antony, J.; Ehrlich, S.; Krieg, H. A Consistent and Accurate Ab Initio Parametrization of Density Functional Dispersion Correction (DFT-D) for the 94 Elements H-Pu. *J. Chem. Phys.* **2010**, *132* (15).

(5) Grimme, S.; Hansen, A.; Brandenburg, J. G.; Bannwarth, C. Dispersion-Corrected Mean-Field Electronic Structure Methods. *Chem. Rev.* **2016**, *116* (9), 5105–5154.

(6) Grimme, S.; Ehrlich, S.; Goerigk, L. Software News and Updates Gabedit — A Graphical User Interface for Computational Chemistry Softwares. *J. Comput. Chem.* **2011**, 1456–1464.

(7) Dovesi, R.; Erba, A.; Orlando, R.; Zicovich-Wilson, C. M.; Civalleri, B.; Maschio, L.; Rérat, M.; Casassa, S.; Baima, J.; Salustro, S.; Kirtman, B. Quantum-Mechanical Condensed Matter Simulations with CRYSTAL. *Wiley Interdiscip. Rev. Comput. Mol. Sci.* **2018**, *8* (4), 1–36.

(8) Dovesi, R.; Saunders, V. R.; Roetti, C.; Orlando, R.; Zicovich-Wilson, C. M.; Pascale, F.; Civalleri, B.; Doll, K.; Harrison, N. M.; Bush, I. J.; D'Arco, P.; Llunell, M.; Causà, M.; Noël, Y.; Maschio, L.; Erba, A.; Rerat, M.; Casassa, S. *CRYSTAL17 User's Manual*; University of Torino: Torino, 2017.

(9) Becke, A. D. Density-Functional Thermochemistry. III. The Role of Exact Exchange. *J. Chem. Phys.* **1993**, *30*, 5648–5652.

(10) Lee, C.; Yang, W.; Parr, R. G. Development of the Colle-Salvetti Correlation-Energy Formula into a Functional of the Electron Density. *Phys. Rev. B* **1988**, *37*, 785–789.

(11) Vosko, S. H.; Wilk, L.; Nusair, M. Accurate Spin-Dependent Electron Liquid Correlation Energies for Local Spin Density Calculations: A Critical Analysis. *Can. J. Phys.* **1980**, *58* (8), 1200–1211.

(12) Monkhorst, H. J.; Pack, J. D. Special Points for Brillouin-Zone Integrations. *Phys. Rev. B* **1976**, *13*, 5188–5192.

(13) Peintinger, M. F.; Oliveira, D. V.; Bredow, T. Consistent Gaussian Basis Sets of Triple-Zeta Valence with Polarization Quality for Solid-State Calculations. *J. Comput. Chem.* **2013**, *34* (6), 451–459.

(14) Vilela Oliveira, D.; Laun, J.; Peintinger, M. F.; Bredow, T. BSSE-Correction Scheme for Consistent Gaussian Basis Sets of Double- and Triple-Zeta Valence with Polarization Quality for Solid-State Calculations. *J. Comput. Chem.* **2019**, *40* (27), 2364–2376.

(15) Laun, J.; Vilela Oliveira, D.; Bredow, T. Consistent Gaussian Basis Sets of Double- and Triple-Zeta Valence with Polarization Quality of the Fifth Period for Solid-State Calculations. *J. Comput. Chem.* **2018**, *39* (19), 1285–1290.

(16) Schäfer, A.; Horn, H.; Ahlrichs, R. Fully Optimized Contracted Gaussian Basis Sets for Atoms Li to Kr. *J. Chem. Phys.* **1992**, *97* (4), 2571–2577.

(17) Weigend, F.; Ahlrichs, R. Balanced Basis Sets of Split Valence, Triple Zeta Valence and Quadruple Zeta Valence Quality for H to Rn: Design and Assessment of Accuracy. *Phys. Chem. Chem. Phys.* **2005**, *7* (18), 3297–3305.

(18) Pascale, F.; Zicovich-Wilson, C. M.; Lopez Gejo, F.; Civalleri, B.; Orlando, R.; Dovesi, R. The Calculation of the Vibrational Frequencies of Crystalline Compounds and Its Implementation in the CRYSTAL Code: Crystalline Compounds and the CRYSTAL Code. *J. Comput. Chem.* **2004**, *25*, 888–897.

(19) Zicovich-Wilson, C. M.; Pascale, F.; Roetti, C.; Saunders, V. R.; Orlando, R.; Dovesi, R. Calculation of the Vibration Frequencies of α-Quartz: The Effect of Hamiltonian and Basis Set. *J. Comput. Chem.* **2004**, *25* (15), 1873–1881.
(20) Resta, R. Polarization as a Berry Phase. *Europhys. News* **1997**, *28* (1), 18.
(21) Dall'Olio, S.; Dovesi, R.; Resta, R. Spontaneous Polarization as a Berry Phase of the Hartree-Fock Wave Function: The Case of $KNbO_3$. *Phys. Rev. B* **1997**, *56* (16), 10105–10114.
(22) Resta, R. Macroscopic Polarization in Crystalline Dielectrics: The Geometric Phase Approach. *Rev. Mod. Phys.* **1994**, *6*, 899–915.
(23) Hinuma, Y.; Pizzi, G.; Kumagai, Y.; Oba, F.; Tanaka, I. Band Structure Diagram Paths Based on Crystallography. *Comput. Mater. Sci.* **2017**, *128*, 140–184.
(24) Beata, G.; Perego, G.; Civalleri, B. CRYSPLOT: A New Tool to Visualize Physical and Chemical Properties of Molecules, Polymers, Surfaces, and Crystalline Solids. *J. Comput. Chem.* **2019**, *40*, 2329–2338.
(25) Williams, T; Kelley, C. et al. Gnuplot 5.4.5 (October 2022): an interactive plotting program. http://www.gnuplot.info/
